# Hepatitis C virus 3′UTR regulates viral translation through direct interactions with the host translation machinery

**DOI:** 10.1093/nar/gkt543

**Published:** 2013-06-19

**Authors:** Yun Bai, Kaihong Zhou, Jennifer A. Doudna

**Affiliations:** ^1^Department of Molecular and Cell Biology, University of California, Berkeley, CA 94720, USA, ^2^Howard Hughes Medical Institute, University of California, Berkeley, CA 94720, USA, ^3^Department of Chemistry, University of California, Berkeley, CA 94720, USA and ^4^Physical Biosciences Division, Lawrence Berkeley National Laboratory, Berkeley, CA 94720, USA

## Abstract

The 3′ untranslated region (3′UTR) of hepatitis C virus (HCV) messenger RNA stimulates viral translation by an undetermined mechanism. We identified a high affinity interaction, conserved among different HCV genotypes, between the HCV 3′UTR and the host ribosome. The 3′UTR interacts with 40S ribosomal subunit proteins residing primarily in a localized region on the 40S solvent-accessible surface near the messenger RNA entry and exit sites. This region partially overlaps with the site where the HCV internal ribosome entry site was found to bind, with the internal ribosome entry site-40S subunit interaction being dominant. Despite its ability to bind to 40S subunits independently, the HCV 3′UTR only stimulates translation *in cis*, without affecting the first round translation rate*.* These observations support a model in which the HCV 3′UTR retains ribosome complexes during translation termination to facilitate efficient initiation of subsequent rounds of translation.

## INTRODUCTION

Synergistic interactions between the 5′cap structure and the 3′poly(A) tail are crucial for efficient translation of cellular messenger RNAs (mRNAs) (1,2). Such end-to-end synergy has also been reported for various RNA viruses. In some plant viruses, 3′-cap-independent translation elements are thought to bind and deliver translation initiation factors to the 5′end of the viral genome through long-range RNA–RNA contacts, thereby stimulating translation initiation (3–6). Similarly, the conserved 3′ untranslated regions (3′ UTRs) of animal RNA viruses including classical swine fever virus (7), foot-and-mouth disease virus (8), dengue virus (9) and hepatitis C virus (HCV) (10) have been reported to regulate viral translation. However, in these cases, the molecular mechanisms by which the 3′UTRs function remain to be elucidated.

**Figure 1.**
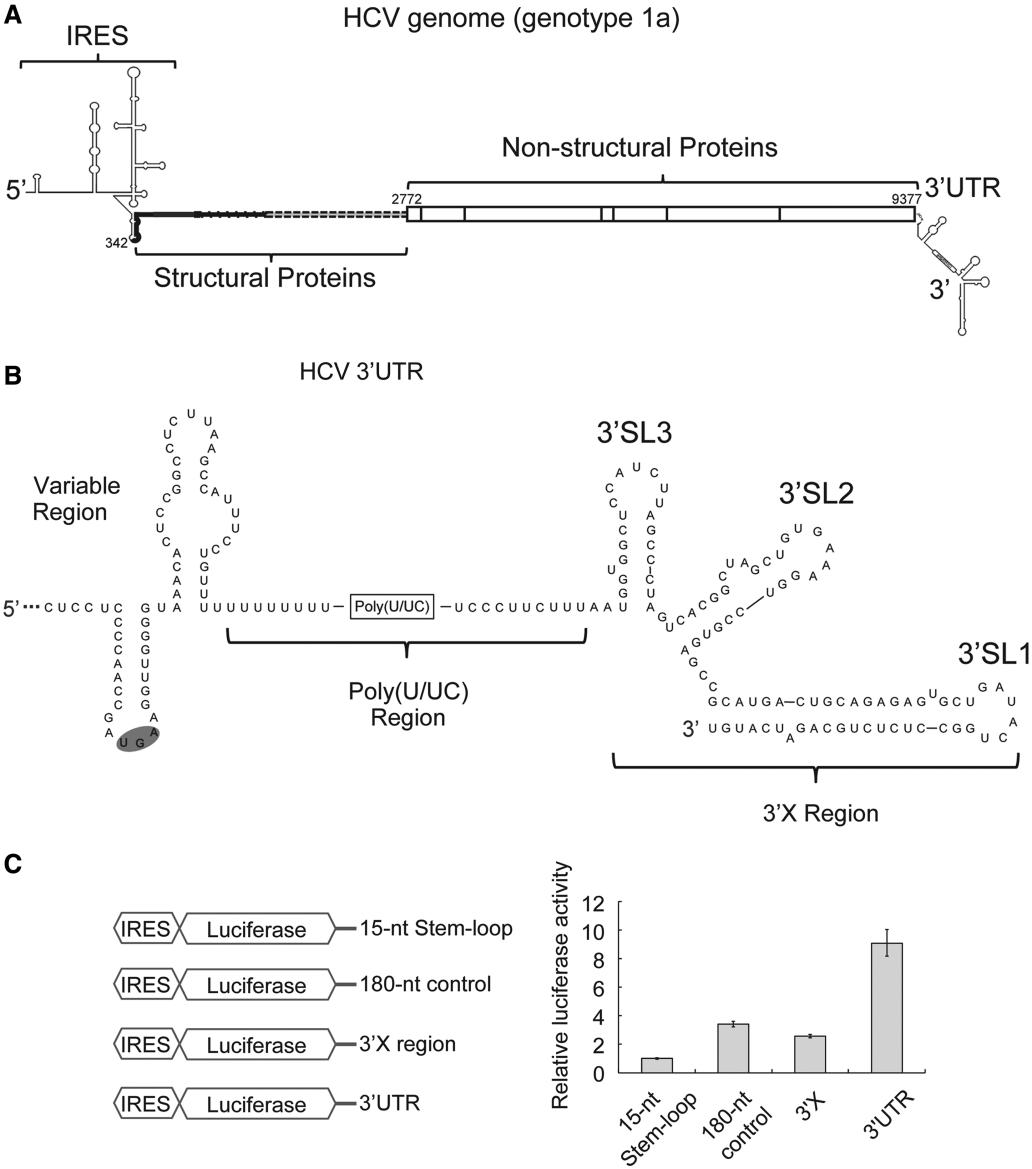
The HCV 3′UTR stimulates IRES-dependent translation in cell culture. (**A**). Schematic drawing of the HCV genome. Secondary structures of both UTRs are indicated. The coding region is shown in thick lines for structural proteins and in boxes for non-structural proteins. Numbers are labeled according to genotype 1a strain H77 (**B**). Secondary structure of the 3′UTR of HCV genotype 1a. The variable region, poly(U/UC) region and three-stem loops in the 3′X region are labeled (**C**). Schematic drawing of different constructs used in translation assays are shown on the left. In the control construct, a 15-nt stem-loop (CUGCCGUAUAGGCAG) was attached to the 3′end the luciferase mRNA via a 5-nt linker (GUUCA) to ensure mRNA stability. The 180-nt control sequence is adopted from the pUC19 vector (447–630). On the right shows luciferase activities from cell-based translation assays using different RNA constructs. Luciferase activities in all experiments are normalized against that of the construct with a 15-nt stem loop downstream of the luciferase mRNA.

In HCV, a formidable disease that infects >170 million people worldwide, the viral RNA includes a ∼220 nt conserved 3′UTR (Figure 1A). The 3′UTR features a variable region, followed by a poly(U/UC) tract and a 3′X region with three predicted stem loops (3′SL1-3) (Figure 1B). Along with the HCV 5′UTR, which includes an internal ribosome entry site (IRES) (Figure 1A), the highly conserved 98-nt 3′X region is critical for viral replication (11). Both the variable region sequence and the length of the poly(U/UC) tract in the 3′UTR vary widely among different viral genotypes but are conserved within the same genotype.

HCV translates its genome by hijacking the host translation machinery through its IRES. The IRES interacts directly with and induces a conformational change in the 40S subunit, ensuring correct positioning of the HCV open reading frame in the 40S mRNA binding cleft. Subsequently, eukaryotic translation initiation factor 3 (eIF3) and Met-tRNA_i_-eIF2 are recruited to the complex, facilitated by the IRES-eIF3 interaction (12,13). Formation of the 48S initiation complex triggers guanosine-5′-triphosphate (GTP) hydrolysis to promote joining of the 60S subunit. The fully assembled 80S ribosome then proceeds to translation elongation (14). This IRES-dependent translation initiation process has been extensively studied. On the contrary, mechanistic understanding of the functional roles of the HCV 3′UTR in translation is limited. Despite the observation that the 3′UTR is not required for IRES-dependent translation, several studies implicated this region in translation stimulation (10,15–18). Other studies, however, suggested that the same region either has no effect on (19) or inhibits translation (20). We show here that the HCV 3′UTR interacts directly with both the 40S ribosomal subunit and eIF3 to stimulate viral translation *in cis*. Results of functional assays, cross-linking data and interaction mapping support a model in which the HCV 3′UTR enhances translation by transferring components of the translation machinery from the 3′ to the 5′ end of viral mRNA, especially at the termination stage of one round of translation.

## MATERIALS AND METHODS

### Constructs cloning

Plasmids for transcription of target RNAs were generated by insertion of sequences of interest into the EcoRI/BamHI site of pUC19 vector. Constructs for *in vitro* and cell-based translation assays were made by inserting different fragments of HCV 3′end into a previously reported construct containing MS2-IRES-Luciferase (21). Constructs for recombinant eIF3 8-subunit core complex were a gift from Jamie Cate’s laboratory (22).

### RNA transcription, purification and end labeling

RNAs used in this study were transcribed *in vitro* using T7 polymerase. Reactions were ethanol precipitated and further purified by denaturing polyacrylamide gel electrophoresis. The 5′ end radiolabeled RNA was generated using [γ-P^32^]-ATP and T4 polynucleotide kinase according to standard protocol, followed by further purification using denaturing polyacrylamide gel electrophoresis. The 3′ end fluorescently labeled RNA was made using Fluorescein-5-Thiosemicarbazide (Life technologies/Molecular Probes) as described (22).

### Purification of the 40S ribosomal subunit and eIF3

The 40S subunit from rabbit reticulocyte lysate (RRL; Green Hectares) was isolated as described (23,24). Human 40S subunit and native eIF3 was purified from HeLa cytoplasmic lysate according to a previous report (25). Recombinant eIF3 8-subunit core complex was expressed in *Escherichia coli* strain Rosetta2(DE3)pLysS and purified using an established protocol (22).

### Binding reactions

RNAs were annealed by heating at 65°C for 2 min then cooled down to room temperature in folding buffer [20 mM Hepes (pH 7.5), 150 mM KCl, 5 mM MgCl_2_]. Because the 40S subunit tends to bind RNA non-selectively, it is difficult to find a suitable competitor that not only blocks non-specific interactions but also leaves all specific 3′UTR-40S contacting surfaces uncovered. Therefore, we used the same binding condition as used for studying the interactions between the HCV IRES and the 40S subunit (24). For simple binding assays, radiolabeled RNAs were mixed with various concentrations of the 40S subunit, eIF3 or the 40S-eIF3 complex in binding buffer [20 mM Hepes (pH 7.5), 100 mM KAc, 200 mM KCl, 3 mM MgCl_2_, 1 mM Tris(2-carboxyethyl)phosphine hydrochloride (TCEP)]. For competition assays, unlabeled competitor RNAs of different concentrations were mixed with 10 nM of the 40S subunit before addition of radiolabeled RNAs. All binding and competition reactions were incubated at 37°C for 20 min to allow complex formation. The 40-nt mRNA mimicker used in the competition assay was chemically synthesized (5′-GAAUCUCGCUCAUGGUCUCUCUCUCUCUCUCUCUCUCUCU-3′, from IDT). Reactions were then analyzed by either electrophoretic mobility shift assays (EMSA) or filter binding assays, visualized by phosphorimaging and quantified using ImageQuant (GE healthcare). The apparent *K*_d_ was calculated by fitting the binding data with a standard binding isotherm described by the equation: θ = [P] / ([P] + *K*_d_) using Kaleidagraph (Synergy Software), where θ is the fraction of RNA bound, [P] is the concentration of either the 40S subunit, eIF3, or the 40S-eIF3 complex.

### Selective 2′-hydroxyl acylation analyzed by primer extension analysis

Selective 2′-hydroxyl acylation analyzed by primer extension (SHAPE) analysis was performed using an RNA containing 3′UTR-Δ3′SL1 and a 3′ handle (5′-CCGAUCCGCUUCGGCGGAUCCAAAUCGGGCUUCGGUCCGGUUC-3′). The SHAPE reactions were conducted according to previous report (26) with small modifications. The reaction buffer was the same as the binding buffer described earlier in the text, and 40 mM of benzoyl cyanide (BzCN) (Sigma) was used as the 2′ hydroxyl-selective electrophile instead. The short half-life of BzCN in aqueous solution makes it suitable for targeting transient interactions (27). Raw traces from fragment analysis were analyzed using ShapeFinder (28).

### *In vitro* and cell-based translation assays

*In vitro* translation assays were preformed using nuclease treated RRL (Promega) or nuclease-treated HeLa cytoplasmic extract. A 15 μl of RRL-based reaction system containing 56% (v/v) RRL, supplemented with 20 μM amino acids, 1.3 U/μl RNasin Plus RNase inhibitor (Promega), Complete Protease Inhibitor Cocktail according to manual (Roche), 2 mM DTT, 1.8 mM MgCl_2_, 45 mM KCl and 26 mM KOAc (29). A 15 ul of HeLa-based reaction system was prepared as described. In all, 50 ng of mRNA was used in each reaction. For experiments with the 3′UTR RNA *in trans*, 50 ng of that RNA was also included. For cell-based translation assay, Huh-7 cells were grown and transfected following standard protocol (16). Two hours post-transfection, cells were harvested, followed by treatment with either Passive Lysis buffer (Promega) for measurement of luciferase activity or TRIzol (Life technologies) for total RNA extraction. For both *in vitro* and cell-based assays, luciferase activity was measured using luciferase assay system (Promega) on a GloMax 96 Microplate Luminometer. Relative RNA levels were measure by reverse transcription of luciferase RNA using SuperScript III reverse transcriptase (Life technologies) followed by quantitative RT-PCR using SYBR Green PCR master mix (Life technologies) on a ABI 7900 HT instrument. For continuous *in situ* measurement of luciferase activity, 0.1 mM luciferin as well as 2 mM of ATP were included in the translation mixture derived from RRL and HeLa cytoplasmic extract as stated earlier in the text. From 20 s after the addition of mRNA, luciferase activity was measured every second at 37°C for 1000 or 1800 s using a GloMax 20/20 single tube luminometer.

### 4-thiouridine mediated UV-cross-linking

The 4-thiouridine-5′-triphosphate (Trilink biotechnologies) were randomly incorporated into the HCV 3′UTR in 1:20 ratio by *in vitro* transcription. The 4-thiouridine-5′-triphosphate body labeled HCV 3′UTR was subsequently 3′end labeled with fluorescein, so that it can be distinguished from RNAs from other sources in later gel analysis. RNA annealing and complex formation were performed under the same conditions as for the binding assays. Complex was exposed to 365 nm UV light for 15 min, using a cool metal block to prevent the samples from overheating. Reactions were concentrated and heated in the presence of denaturing SDS–PAGE loading dye before applied to a SDS–PAGE gel. Gels were later visualized both on a Typhoon Imager for fluorescent signal of HCV 3′UTR RNA and by staining with coomassie blue for protein signal. Target bands were then extracted for liquid chromatography-tandem mass spectrometry (LC-MS/MS) analysis.

### Sucrose gradient analysis

Sucrose gradient analysis was performed using 3′end fluorescently labeled 3′UTR either alone or in complex with the ribosome. For free RNA and the 3′UTR-40S complex, samples were applied to a 5–20% sucrose gradient and centrifuged in a Beckman SW41 rotor at 40 000 rpm for 3.5 h. For 3′UTR-80S complex, to better visualize the 80S ribosome peak, sample was applied to a 10–50% sucrose gradient and centrifuged in Beckman SW41 rotor at 25 000 rpm for 12 h. For the analysis of *in vitro* translation reactions, samples were applied to a 10–40% sucrose gradient and centrifuged in Beckman SW41 rotor at 40 000 rpm for 3.5 h. Gradients were fractionated using ISCO UV detector and position of either the 3′UTR or reporter mRNA were determined by analyzing fractions on a denaturing polyacrylamide gel, which was later visualized using Typhoon Imager.

### Determination of the first round of translation rate and the steady state luciferase production rate

The first round translation time and the steady state luciferase production rate were calculated as reported (30) with slight modifications. The smoothed first derivative of the luciferase activity was fitted to a cumulative Gaussian distribution using the program Prism 5 (GraphPad) to the equation:

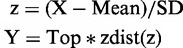



The Mean value of the fitted reflects the T_1st_ and the Top value reflects the Max (steady state luciferase production).

## RESULTS

### The HCV 3′UTR stimulates IRES-dependent translation in cell culture

To elucidate the role of the HCV 3′UTR in IRES-dependent translation, we first examined how it modulates translation in cell culture using the human hepatoma cell line Huh-7. Different truncations of the HCV 3′end were inserted downstream of firefly luciferase mRNA driven by the HCV IRES (Figure 1C). Two hours post transfection of *in vitro* prepared mRNAs, we found the full-length HCV 3′UTR stimulated translation of luciferase by ∼10-fold (Figure 1C). This stimulation is not due to RNA stabilization, based on measurements of relative mRNA levels by quantitative RT-PCR (Supplementary Figure S1). Moreover, this simulation is more significant than that introduced by an unrelated sequence of similar length adopted from the pUC19 vector (Figure 1C), indicating this enhancement is 3′UTR-sequence specific. In contrast, the highly conserved 3′X region stimulated translation more moderately, by ∼2.5 fold (Figure 1C), consistent with a previous report showing that deletion of the 3′X region from the HCV 3′UTR reduced translation by ∼2-fold (31). The degree of this 3′X-mediated stimulation is similar to that observed using the unrelated sequence (Figure 1C), indicating this could be a sequence-independent effect. Although it is not clear whether the 3′X region alone functions specifically to stimulate translation, any such effect is markedly reduced relative to the effect of the full-length 3′UTR. Therefore, either sequences outside of the 3′X region or the entire 3′UTR appear to be required for robust translation stimulation.

### The HCV 3′UTR interacts directly with the 40S ribosomal subunit

**Figure 2.**
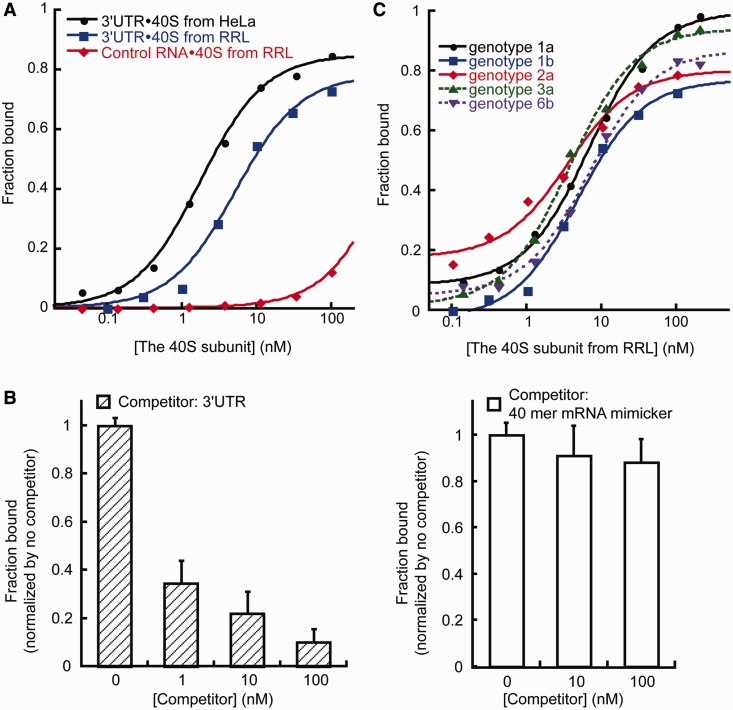
The HCV 3′UTR exhibits strong, specific and conserved interaction with the 40S subunit. (**A**). Binding isotherms for the 3′UTR-40S interaction. The HCV 3′UTR interacts with the 40S subunits isolated from both HeLa extract and RRL, whereas an RNA of similar size, corresponding to part of the HCV NS5B protein-coding region directly upstream of the 3′UTR (nucleotides 9184–9386 from HCV strain H77) showed no obvious interaction. (**B**). The 3′UTR-40S interaction is subject to competition by unlabeled 3′UTR competitor (left panel). The 3′UTR-40S interaction is not subject to competition by a random 40-nt mRNA mimicker (right panel). The amount of competitor used is indicated. The bound fraction shown has been normalized. (**C**). Binding isotherm of the HCV 3′UTR from different genotypes binding to the 40S subunit. The *K*_d_ values are 6.7 ± 0.7 nM (genotype 1a), 4.7 ± 0.9 nM (genotype 1b), 3.5 ± 1 nM (genotype 2a), 3.7 ± 0.4 nM (genotype 3a), 6.7 ± 0.7 nM (genotype 6b).

The HCV IRES interacts directly with the host 40S ribosomal subunit to correctly position the start codon of viral mRNA into the P site for initiation of translation. This interaction between the HCV IRES and the 40S subunit can happen in both the presence and absence of initiation factors (14,24,32). As the HCV 3′UTR was observed to stimulate translation, we asked whether it can interact with the 40S subunit as well. Initial results from EMSA conducted using 40S subunit samples purified from both HeLa cytoplasmic extract, and RRL provided evidence for such a direct interaction (Figure 2A). The apparent *K*_d_ for the 3′UTR-40S interaction (1.8 ± 0.2 nM and 5.4 ± 0.8 nM for 40S subunit from HeLa and RRL, respectively) (Figure 2A) is similar to that determined for the HCV IRES-40S subunit interaction (∼2 nM) measured under the same salt and buffer conditions (24). As the 3′UTR binds to human and rabbit 40S subunits with similar low nanomolar affinities, these two types of 40S samples were used interchangeably in subsequent biochemical experiments.

We next evaluated the specificity of this observed 3′UTR-40S interaction. An RNA of similar length corresponding to part of the HCV NS5B protein-coding region directly upstream of the 3′UTR showed no detectable interaction with the 40S subunit under the same conditions (Figure 2A). In addition, the 3′UTR-40S interaction is subject to competition by unlabeled 3′UTR (Figure 2B). Although the 3′UTR could bind to the *E.coli* 70S ribosome, this interaction was lost on addition of competitor yeast tRNA, whereas the 3′UTR-40S interaction was retained in the presence of competitor tRNA (Supplementary Figure S2A). These observations indicate that the 3′UTR-40S interaction is specific. Because of the ssRNA binding ability of the 40S subunit, we also tested whether binding to the single-stranded poly(U/UC) stretch of the 3′UTR accounted for the observed interaction. However, a 40-nt transcript that binds the mRNA binding cleft of the 40S subunit (25) did not compete with the 3′UTR-40S interaction (Figure 2B). Furthermore, a 25-nt oligo(U) mimicking part of the poly(U/UC) region also did not compete with the 3′UTR for 40S subunit binding (Supplementary Figure S2B). Based on these results, we concluded that the HCV 3′UTR is capable of making a strong yet specific interaction with regions of the 40S subunit outside of the mRNA binding cleft.

### All regions of the HCV 3′UTR are required for 40S binding

We tested the 3′UTRs from several other genotypes of HCV to investigate whether the observed 3′UTR-40S interaction is conserved. Using a similar EMSA, all HCV 3′UTRs examined, including those from genotypes 1a, 1b, 2a, 3a and 6b, were found to form a high-affinity complex with the 40S subunit. Their measured binding affinities to the 40S subunit are all within the low nanomolar range (3.5 ± 1 nM to 6.7 ± 0.7 nM, Figure 2C), and these different 3′UTRs can efficiently compete with each other for 40S interaction (Supplementary Figure S2C).

**Figure 3.**
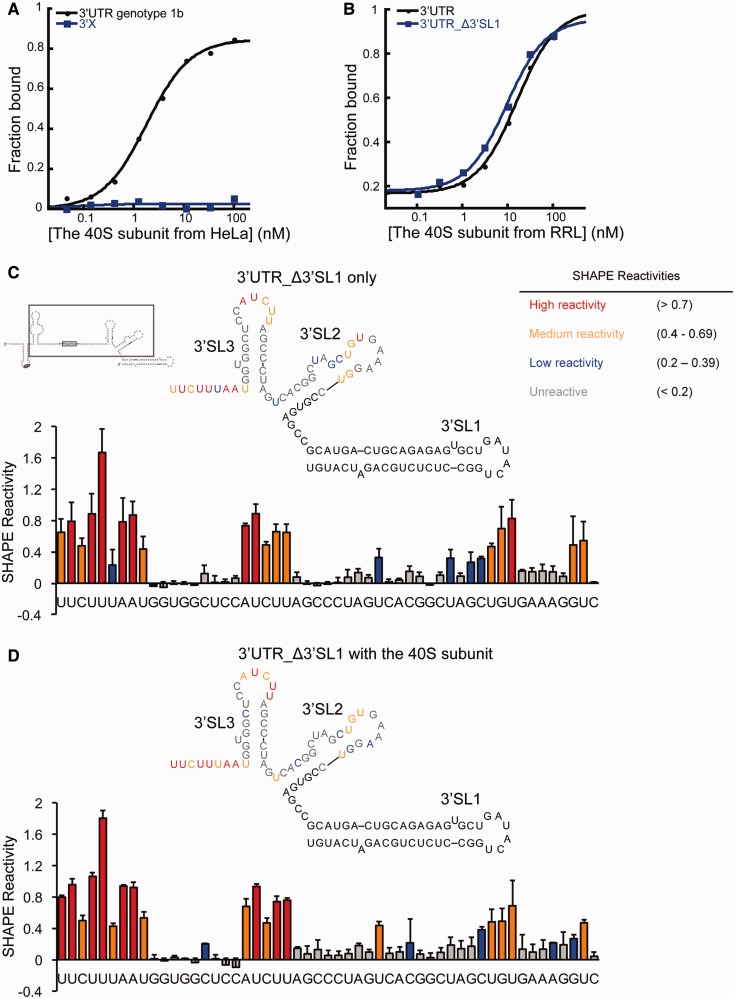
The contribution of the 3′X region in the HCV 3′UTR-40S interaction (**A**). Binding isotherms showing the 3′X region alone cannot interact with the 40S subunit. (**B**). Binding isotherms showing full-length 3′UTR and 3′UTR_Δ3′SL1 interact with the 40S subunit similarly. (**C** and **D**). SHAPE analysis of the 3′UTR either alone or in complex with the 40S subunit. The gray box shows the construct used in these experiments. In red are highly reactive nucleotides, in orange are medium reactive nucleotides, in blue are nucleotides with low reactivity, and in gray are unreactive nucleotides. Nucleotides either with no SHAPE data or not included in the construct are shown in black.

As all HCV 3′UTRs share the highly conserved 3′X region (Supplementary Figure S2D), a tempting possibility was that the 3′X region mediates the 3′UTR-40S interaction. However, subsequent binding assays revealed no significant interaction between the isolated 3′X region and the 40S subunit (Figure 3A). Deletion of the entire 3′X region made the rest of the 3′UTR RNA, which ends with a long poly(U/UC) stretch, highly subject to degradation. However, truncation of 3′SL1 (3′UTR_Δ3′SL1), which covers half of the 3′X region, had no impact on the 3′UTR-40S interaction (Figure 3B).

We next asked whether the 3′X region contributes significantly to the interaction with the 40S subunit in the context of full-length 3′UTR. To answer this question, SHAPE chemistry (33) was used to analyze the flexibility of nucleotides in the 3′X region in both the presence and absence of the 40S subunit. The 3′UTR_Δ3′SL1 RNA was used here because it binds the 40S subunit with similar affinity to that of the full-length 3′UTR, yet lacks the stable 3′SL1 hairpin that blocks primer extension-based detection of chemical modification sites. In the absence of the 40S subunit, the SHAPE reactivity of nucleotides in the 3′X region is consistent with the predicted secondary structure. Four consecutive nucleotides in the 6-nt loop of 3′SL2 showed either low or no reactivity, suggesting their involvement in tertiary structure formation (Figure 3C). In the presence of the 40S subunit, only minor changes in SHAPE reactivity were observed overall. Nucleotides showing these changes are localized to 3′SL2; the remainder of the 3′X region showed no significant SHAPE reactivity change on binding to the 40S subunit (Figure 3C and D). Therefore, the 3′X region does not appear to make significant contributions to the 3′UTR-40S interaction. However, these results also suggest that 3′SL2 could make weak contacts with the 40S subunit, which may help the positioning of the 3′UTR.

As the SHAPE results suggested that the 3′X region alone is insufficient to mediate a tight interaction with the 40S subunit, we then asked what role other regions of the 3′UTR play in interacting with the 40S subunit. The SHAPE assay was not applicable here because the poly(U/UC) tract led to stops in primer extension. However, earlier experiments showed that a 25-nt oligo(U) can not compete with the 3′UTR-40S interaction (Supplementary Figure S2B), indicating the poly(U/UC) tract alone is likely not sufficient for binding. In light of these results, we conclude that all regions of the 3′UTR are required for the interaction with the 40S subunit through a combination of direct and indirect contacts to the translation machinery.

### The HCV 3′UTR binds to proteins on the solvent side of the 40S subunit

**Figure 4.**
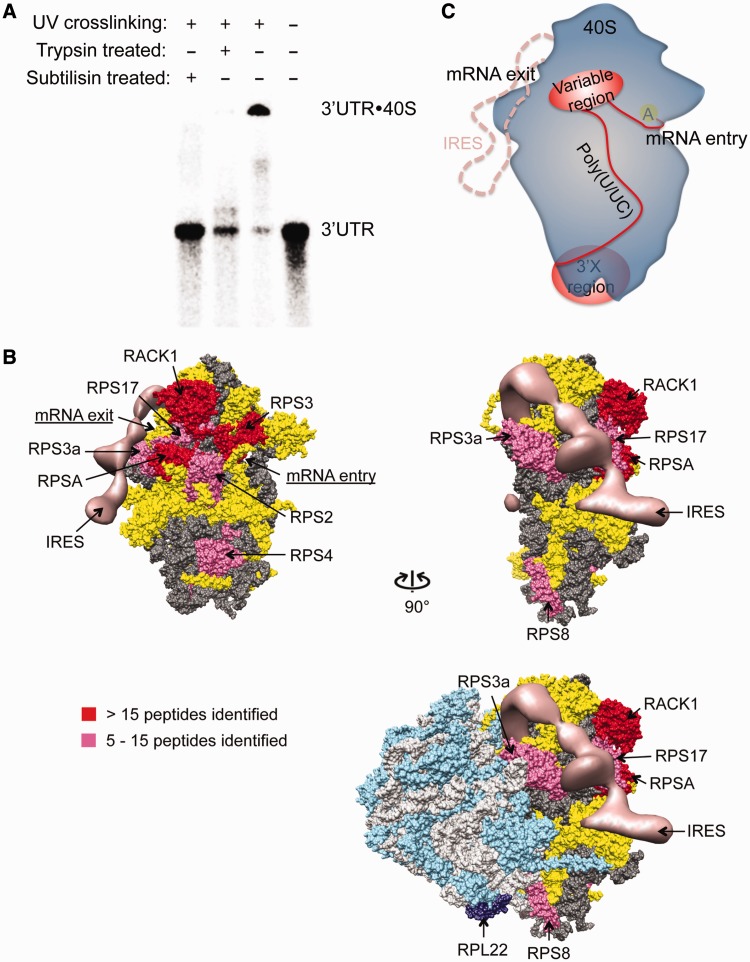
Mapping of the 3′UTR interacting region on the 40S subunit. (**A**). Non-specifically cross-linked 3′UTR-40S complex can be degraded to the 3′UTR RNA alone by both trypsin and subtilisin. (**B**). Mapping result from 4-thiouridine mediated cross-linking of the 3′UTR to the 40S subunit. The hits were categorized based on the total number of spectra observed. Proteins with >15 spectra identified are assigned as strong binders (red), whereas those with between 5 and 15 spectra are assigned as moderate binders (magenta). Entries with four or less spectra are considered background noise from non-specific cross-linking. The HCV IRES is shown in salmon. In dark and light gray are ribosomal RNAs of the 40S and the 60S subunit, respectively. In yellow are the 40S ribosomal proteins not interacting with the 3′UTR. In cyan and dark purple are the 60S ribosomal proteins with RPL22 labeled in dark purple, which was indicated to interact with the 3′X region (34). All the 3′UTR interacting ribosomal/ribosome associated proteins are labeled. (**C**). Binding model for the HCV 3′UTR and the 40S ribosome. In blue is the 40S subunit, with position of the A site indicated by the yellow oval. In red is the 3′UTR with the variable and the 3′X region shown in oval and the poly(U/UC) tract as well as the linker between the stop codon and the beginning of the variable region stem-loop shown as curved lines. The HCV IRES-binding position on the 40S ribosome is shown in salmon dash line.

To determine where the HCV 3′UTR binds on the 40S ribosomal subunit, we first asked whether the interaction is mediated by ribosomal RNA, ribosomal proteins or both. Protease treatment of the 3′UTR-40S complex, which was cross-linked by exposure to UV light, led to a product of reduced molecular weight that had the same electrophoretic mobility as the 3′UTR by itself (Figure 4A). This implies that the HCV 3′UTR interacts predominantly with ribosomal proteins rather than ribosomal RNA.

To map the HCV 3′UTR contact points on the 40S subunit, we used a 4-thiouridine-mediated cross-linking strategy (35). In addition to the poly(U/UC) tract, there are uridines in all the single-stranded regions except a small bulge in 3′SL1, which was demonstrated earlier to be dispensable for the 3′UTR-40S interaction. After cross-linking the 40S subunit sample with fluorescently labeled 3′UTR RNA, a slowly migrating species that contained both fluorescently labeled 3′UTR and protein components was detected by denaturing gel electrophoresis. This complex, observed only in the presence of both the 3′UTR and the 40S subunit, was isolated and analyzed by mass spectrometry. Data analysis revealed predominant interaction partners to be ribosomal proteins RPS3, RACK1 and RPSA, with additional binding partners including RPS3a, RPS8, RPS4, RPS2 and RPS17 (Table 1) (Supplementary Table S1). Mapping of the homologous proteins on the crystal structure of the yeast ribosome (36) revealed that RPS3, RACK1, RPSA, RPS3a, RPS2 and RPS17 localize to a confined region on the solvent side of the 40S subunit, close to both the mRNA entry and exit sites (Figure 4B). The existence of this defined 3′UTR binding surface underscores the specificity of the 3′UTR-40S interaction. Interestingly, the distance between the A site and the 3′UTR-binding patch can be bridged by ∼10 nt. This raises the possibility that on translation termination, when the HCV stop codon is positioned in the ribosomal A site, the 10 nt between the stop codon and the beginning of the stem-loop in the 3′UTR variable region could enable the variable region to contact the 3′UTR-binding surface of the 40S subunit. If so, such an interaction could explain our observation that the variable region is required for the 3′UTR-40S complex formation.

**Table 1.** Summary of mass spectrometry results

Protein name	Spectrum count	Sequence count
40S ribosomal subunit		
RPS3	27	12
RACK1	18	9
RPSA	17	7
RPS3a	12	6
RPS8	11	6
RPS4, X isoform	10	6
RPS2	7	5
RPS17	5	3
Native eIF3		
eIF3c	13	5
eIF3a	10	2
eIF3b	9	2
eIF3l	2	2
Recombinant eIF3 8 subunit complex		
eIF3c	42	14
eIF3a	41	20
eIF3e	23	13
eIF3l	19	11
eIF3f	10	6
eIF3h	6	4

Ribosomal proteins also interacting with the IRES are underlined.

Two ribosomal proteins that were identified by cross-linking to the HCV 3′UTR in the 40S-bound sample, RPS4 and RPS8, are located outside of the region defined by the other six cross-linked ribosomal proteins. Interestingly, in the 80S ribosome, RPS8 is in close proximity to RPL22, which was proposed to make a weak but specific contact with the 3′X region of HCV (34) (Figure 4B). Therefore, RPS8 may also interact weakly with the 3′X region. Based on these results, we propose that the HCV 3′UTR variable region makes primary contacts to the 40S subunit on the localized binding surface. The poly(U/UC) tract could then span across the 40S subunit, possibly anchored by RPS4, and position the 3′X region to make weak contacts with RPS8 and RPL22 in the context of the 80S ribosome (Figure 4C).

As all the ribosomal proteins identified in the aforementioned experiment remain exposed after joining of the 40S and 60S subunits, we next tested whether the HCV 3′UTR binds to the 80S ribosome. EMSA showed that the HCV 3′UTR interacts with the 80S ribosome with a binding affinity only slightly lower than that with the 40S subunit (11.8 ± 1.5 nM) (Supplementary Figure S3A). The integrity of the 80S ribosome during the course of the binding experiment was confirmed by sucrose-gradient sedimentation. Fluorescently labeled HCV 3′UTR co-migrates with both the 40S subunit and the 80S ribosome, and both migration patterns are distinct from that of the 3′UTR RNA by itself (Supplementary Figure S3B). The small change in binding affinity observed for the 40S versus 80S samples could be due to conformational changes in the binding surface on 60S subunit joining.

### The 3′UTR-40S interaction is disrupted by the IRES but not eIF3

**Figure 5.**
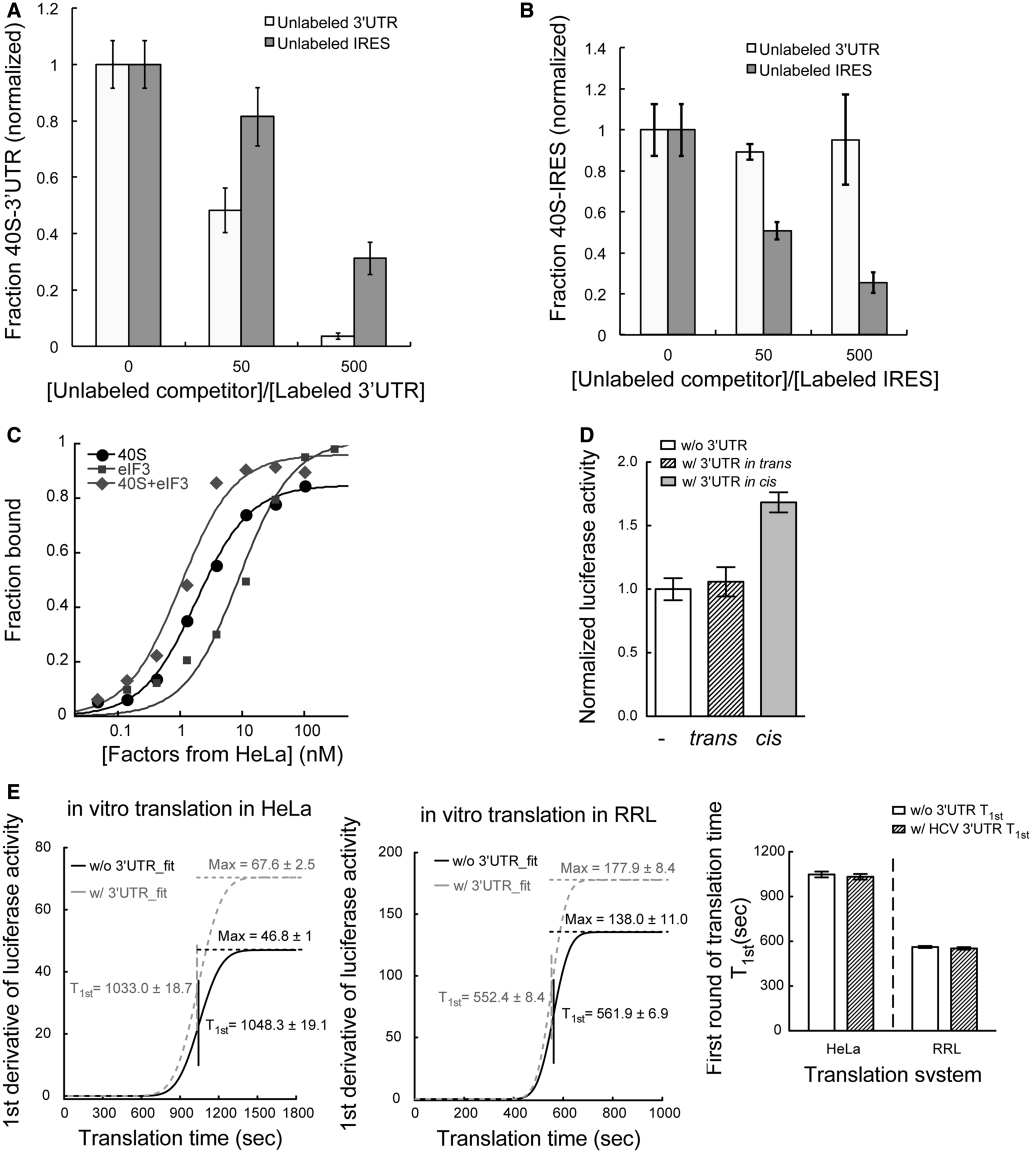
The HCV 3′UTR stimulates translation only *in cis*, without affecting the rate of translation (**A**). Competition assaying showing the 3′UTR-40S interaction is subject to partial competition by unlabeled IRES (dark gray) with the competition less efficient than that from unlabeled 3′UTR (light gray). The competition by 50 times of unlabeled 3′UTR seems modest because the 40S concentration (10 nM) used in these experiment is well above *K*d. (**B**). The IRES-40S interaction is subject to competition by unlabeled IRES (dark gray) but not unlabeled 3′UTR (light gray). The *x*-axis shows the ratio between unlabeled competitor and radiolabeled probe. (**C**). Binding isotherms for the interactions between the HCV 3′UTR and the 40S subunit (*K*_d_ = 1.8 ± 0.1 nM), eIF3 (*K*_d_ = 8.9 ± 2 nM), as well as the 40S-eIF3 complex (*K*_d_ = 1.0 ± 0.2 nM) (**D**). *In vitro* translation assays showing the 3′UTR can only stimulate translation when *in cis* with the IRES and reporter mRNA. Addition of free 3′UTR *in trans* showed no stimulation on IRES-dependent translation. (**E**). First derivative of the real-time recorded luciferase activity was fitted to a cumulative function of normal distribution. Values for both T_1st_ and Max are labeled. For both RRL (left) and Hela lysate (middle) system, presence of the 3′UTR does not affect the T_1st_ but leads to a higher Max value. The bar graph (right) shows the comparison of the T_1st_ for transcripts with and without the HCV 3′UTR in both translation systems.

Comparison of the proposed 3′UTR binding site on the 40S subunit with a cryo-EM model of the HCV IRES-40S subunit complex showed that the 3′UTR and the IRES bind at similar locations (Figure 4B) (37,38). In particular, three of the ribosomal proteins that crosslink to the 3′UTR, RPS2, RPS3 and RPS3a (Table 1), also interact with the IRES (34,39). We wondered whether both RNAs, representing the 5′ and 3′ ends of the HCV mRNA, could bind to the 40S subunit simultaneously. To answer this, we first tested whether the two UTRs show direct interactions. Based on EMSA, we found the mobility of the 3′UTR transcript was only affected at high concentrations (>20 000-fold molar excess) of the HCV IRES; yet, multiple complex species were observed (Supplementary Figure S4). This result, indicating that the interaction between the two UTRs is negligible, is consistent with an earlier report showing that direct RNA–RNA interaction between the two ends of the HCV genome is mediated by the kissing interaction between the IRES and the NS5B protein-coding region (40). Next, we conducted competition binding assays in which pre-mixed IRES-40S or 3′UTR-40S complexes were incubated with a trace amount of radiolabeled 3′UTR or IRES RNA, respectively. The results showed that the HCV IRES partially competed with the 3′UTR for 40S subunit binding (Figure 5A). In the reverse experiment, in which the 3′UTR was tested as a competitor of the IRES-40S interaction, no significant competition was observed (Figure 5B). This observation suggests that the IRES binds to the 40S subunit to form a stable complex that is not subject to 3′UTR competition, whereas the 3′UTR-40S complex is less stable and hence more readily subject to IRES-binding competition.

During translation initiation, the IRES-40S complex recruits the multi-subunit initiation factor eIF3 to form a pre-initiation complex primed for tRNA and 60S subunit binding (12,13). To determine whether the 3′UTR influences this recruitment step, we tested whether the 3′UTR binds to eIF3 and the 40S-eIF3 complex. Nitrocellulose filter binding data showed that the HCV 3′UTR can interact directly with eIF3 (*K*_d_ = 8.9 ± 2 nM) as well as with the 40S-eIF3 complex (*K*_d_ = 1.0 ± 0.2 nM) (Figure 5C). The 3′UTR interacting subunits on eIF3 were then mapped using either native eIF3 purified from HeLa cytoplasmic extract or a reconstituted eIF3 8-subunit core complex (22), using 4-thiouridine mediated cross-linking and mass spectrometry as described earlier (Supplementary Table S2 and S3). With both samples, eIF3a, eIF3c and eIF3l were identified as the most prominent interaction partners. Within the eight-subunit core complex, a few additional subunits were identified, likely because in the intact eIF3 complex those binding surfaces are not exposed (Table 1). Yeast homologues of eIF3a and eIF3c have been reported to interact with homologues of RPSA, RPS3 and RACK1, which are predominant among the 3′UTR-interacting proteins identified on the 40S subunit (41–43). Thus, it is possible that these eIF3 subunits, which are two of the largest proteins in the complex, provide multiple interaction surfaces to bind the ribosome and RNA separately. Therefore, in the 40S-eIF3 complex, the 3′UTR interaction surfaces on both the 40S subunit and eIF3 should be close to each other, making it feasible for both interactions to happen either simultaneously or in a coordinated manner.

### HCV 3′UTR stimulates translation only when *in cis* with the IRES

The binding position of the 3′UTR on the 40S subunit suggests at least two distinct models for its mode of translation stimulation. One possibility is that the 3′UTR enhances the rate of translation initiation by the IRES, possibly by inducing a favorable conformational change in the 40S subunit, which makes it bind more readily to the IRES. Another possibility is that the 3′UTR functions at the end of a round of translation, perhaps by delivering the 40S subunit and initiation factors back to the IRES for subsequent rounds of translation.

The first model predicts that 3′UTR-induced translation stimulation should occur independent of the 3′UTR location within the same transcript as the IRES, whereas the second model instead predicts that stimulation requires the IRES and 3′UTR to co-exist in the same transcript. To evaluate the two proposed models, we asked whether addition of the 3′UTR *in trans* stimulates IRES-dependent translation. Based on *in vitro* translation assays using Hela cytoplasmic extract, free 3′UTR introduced *in trans* in large molar excess to the reporter mRNA did not stimulate IRES-dependent translation (Figure 5D). When the 3′UTR is *in cis* to the mRNA, stimulation of translation was observed in the lysate. The modest stimulation observed *in vitro* is consistent with previous results (10,44) (Figure 5D). To confirm that the free 3′UTR can efficiently interact with the translation machinery in the translation system, we performed sucrose gradient sedimentation experiments and verified that fluorescently labeled free 3′UTR actually co-migrated with the 40S ribosome. This migration pattern is different from that of the 3′UTR by itself (Supplementary Figure S3B and S5A). Therefore, 3′UTR-40S interactions out of the context of a translating mRNA could not stimulate translation. These results pointed to the second model outlined earlier in the text, in which the 3′UTR enhances multiple usage of the translating mRNA by delivering the translation machinery back to the 5′ end of the transcript after each round of protein synthesis. In these *in vitro* assays, the enhancement of translation by the 3′UTR is more moderate comparing with that observed in the cell-based assays, possibly because *in vitro* translation systems are less efficient in supporting multi-round translation.

### HCV 3′UTR promotes multi-round translation without affecting the rate of translation

The proposed model earlier in the text predicts that the rate of the initial round of translation should be the same both in the presence and the absence of the HCV 3′UTR. On the other hand, the mRNA transcript containing the 3′UTR should be more efficient in multi-round translation. This will result in more frequent translation events on the same mRNA molecule, leading to higher levels of protein production from the same molar ratio of transcripts.

To test these predictions, luciferase activity was measured continuously *in situ* over the course of *in vitro* translation reactions using both RRL and HeLa lysate systems. The rate for the first round of translation (T_1st_) was determined according to a previous report (30) with slight modifications, by fitting the first derivative of the luciferase signal to a cumulative distribution function for a normal distribution. The mean of the distribution represents T_1st_, and the maximum (Max) of the curve represents the protein production rate at steady state. Although the absolute translation time was affected by the translation system as well as the quality of the lysate, for both RRL (T_1st_w/ 3UTR_ = 552.4 ± 8.4 s; T_1st_w/o 3UTR_ = 561.9 ± 6.9 s) and HeLa lysate (T_1st_w/ 3UTR_ = 1033.0 ± 18.68 s; T_1st_w/o 3UTR_ = 1048.3 ± 19.1 s) systems, there is no significant differences between the T_1st_ in the presence and absence of the 3′UTR (Figure 5E). Meanwhile, the steady state protein production rates in both lysate systems are higher in the presence of the 3′UTR (RRL: 177.9 ± 8.4; HeLa: 67.6 ± 2.5) than in the absence of the 3′UTR (RRL: 138.0 ± 11.0; HeLa: 46.8 ± 1.0) (Figure 5E). Moreover, we observed that mRNA transcripts with the 3′UTR promote heavier polysome formation when compared with that observed without the 3′UTR (Supplementary Figure S5B). Therefore, the HCV 3′UTR does not affect the rate of a single round of translation. Instead, the enhanced translation is due to efficient multiple rounds of translation on the same mRNA.

## DISCUSSION

**Figure 6.**
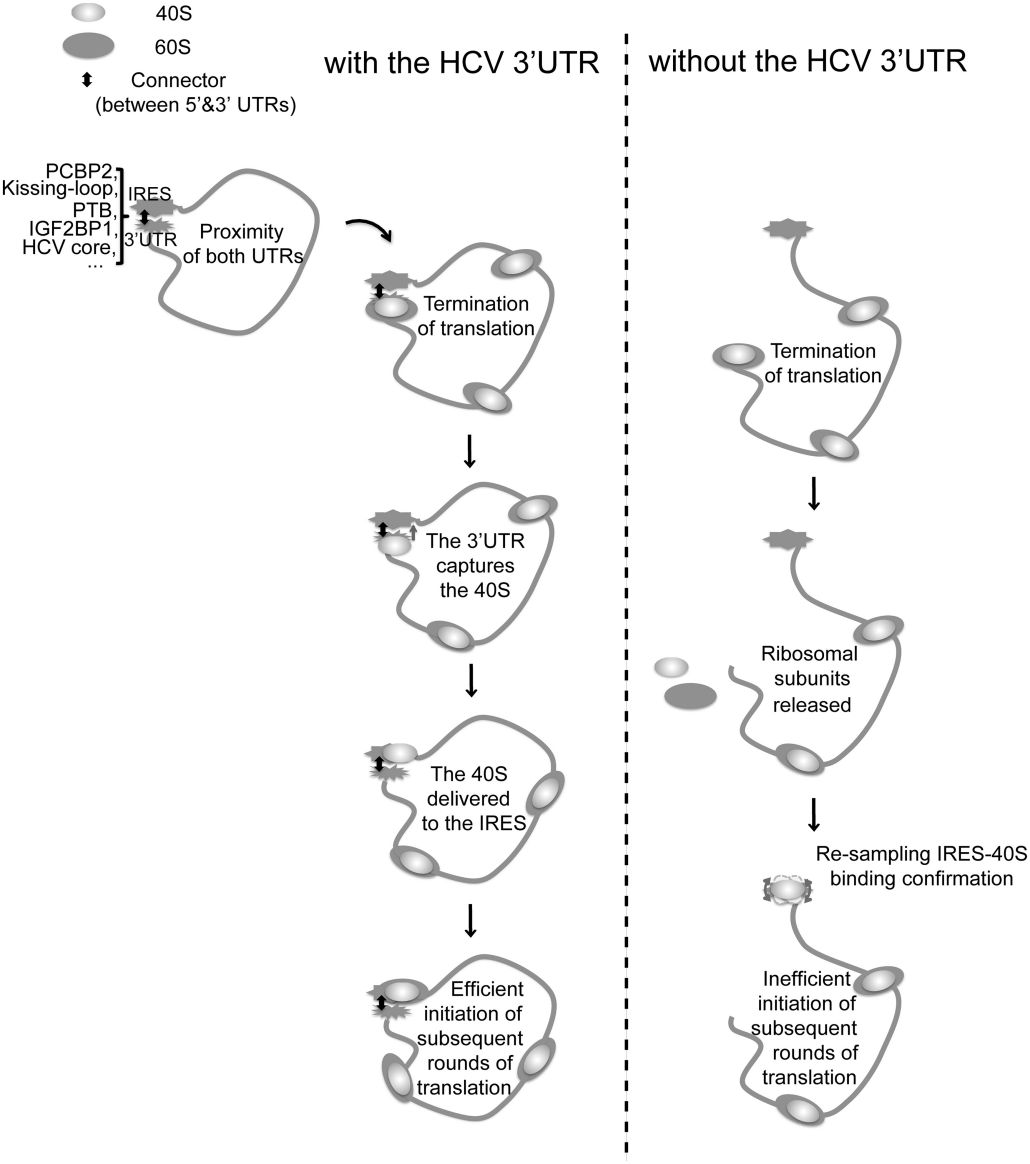
Model of the HCV 3′UTR function in IRES-dependent translation. The two UTRs of HCV are brought to proximity by either long-range RNA–RNA kissing interactions or protein factors (16,17,40,45,46). At the termination stage of translation, when the stop codon is recognized by the A site, the variable region is presented to the 3′UTR binding region on the 40S subunit, promoting the 3′UTR-40S interaction. This interaction retains the 40S subunit after ribosome recycling and transfers it to the IRES in a favorable conformation for effective interactions, which can lead to efficient initiation for subsequence rounds of translation. Without the 3′UTR, for each round of translation, the IRES needs to recruit translation factors from the environment and sample through a variety of binding conformations with the 40S subunit, leading to inefficient initiation of translation.

Based on the aforementioned results, we propose that the HCV 3′UTR stimulates IRES-dependent translation by ‘capturing’ the 40S subunit, and possibly eIF3 as well, and deliver those factors to the IRES for efficient initiation of subsequent rounds of translation (Figure 6). Based on the mapped 3′UTR-binding site on the 40S subunit, at the termination stage of translation, the 40S, either as a separate subunit or as part of the 80S ribosome, can be captured by the 3′UTR. This binding should be highly efficient owing to the high affinity between the HCV 3′UTR and the 40S subunit as well as the high local concentration resulting from the close proximity of the two components. Once the 40S subunit is delivered to the IRES, based on our observation that the IRES-40S interaction is dominant, the ribosomal subunit can be transferred from the 3′UTR to the IRES for the next round of translation.

Consistent with this model, it has been reported that the HCV 3′UTR does not affect the initial 80S ribosome formation (31), and therefore must stimulate translation at a later stage. It has also been reported that the HCV 3′UTR only stimulates cap-dependent translation moderately (∼2-fold) *in vivo*, and this stimulation is observed only in certain cell culture systems (47), emphasizing the requirement of both UTRs for achieving maximum enhancement of translation.

The proposed model implies that the HCV genome forms a closed loop that brings both UTRs adjacent to each other, a possibility supported by several experimental results. The HCV NS5B protein-coding region directly upstream of the 3′UTR has been identified to form long-range kissing interactions with Dom IIId of the IRES as well as the 3′X region of the 3′UTR. The fact that the same region can form interactions with both UTRs indicates the close proximity of the HCV 5′ and 3′UTRs (17,40). In addition, protein factors that regulate HCV translation, including PTB (45), IGF2BP1 (16), PCBP2 (48) and HCV core protein (46), have been shown to interact with both UTRs of HCV (Figure 6). Those interactions can bridge the two ends of HCV and facilitate closed-loop formation. Beyond forming a simple closed loop, these interactions could also restrain the relative orientation of the two UTRs, which could be crucial for efficient delivery of the 40S subunit and eIF3 from the 3′UTR to the IRES at the end of one round of translation for initiation of subsequent rounds of translation.

In conclusion, we identified strong and specific interactions between the HCV 3′UTR and the 40S ribosomal subunit as well as eIF3. These interactions, together with the observations that the two UTRs need to be on the same mRNA transcript for enhanced translation and the 3′UTR does not affect translation rate but promotes polysome formation, support a role for the HCV 3′UTR in capturing components of the translation machinery at the termination stage of translation and delivering those factors to the IRES for efficient multi-round translation. This end-to-end synergy could ensure that only transcripts containing the complete viral genome are translated efficiently. It may also serve as a switch between viral translation and replication such that when HCV replication starts from the 3′end of the viral genome, this synergy between the two ends is interrupted, and viral translation is hence inhibited. Future studies are still required to test these observations and proposed model in the context of real HCV infection.

## SUPPLEMENTARY DATA

Supplementary Data are available at NAR Online: Supplementary Tables 1–3 and Supplementary Figures 1–6.

Supplementary Data
