# Supplementary material for: Hepatitis C virus 3′UTR regulates viral translation through direct interactions with the host translation machinery
Source: Nucleic Acids Res. 2013 Jun 19;41(16):7861–74. doi: 10.1093/nar/gkt543 (PMC3763534; doi:10.1093/nar/gkt543)
Supplement: Supplementary Data [file supp_41_16_7861__index.html]

Hepatitis C virus 3′UTR regulates viral translation through direct interactions with the host translation machinery — Hepatitis C virus 3′UTR regulates viral translation through direct interactions with the host translation machinery — Supplementary Data 

# Hepatitis C virus 3′UTR regulates viral translation through direct interactions with the host translation machinery

## 

files

**Files in this Data Supplement:**

- Supplementary Data - pdf file
- Supplementary Data - xls file
- Supplementary Data - xls file
- Supplementary Data - xls file
