# Supplementary material for: Hepatitis C virus 3′UTR regulates viral translation through direct interactions with the host translation machinery
Source: Nucleic Acids Res. 2013 Jun 19;41(16):7861–74. doi: 10.1093/nar/gkt543 (PMC3763534; doi:10.1093/nar/gkt543)
Supplement: Supplementary Data [file supp_gkt543_nar-01043-r-2013-File009.pdf]

Supplementary Figures

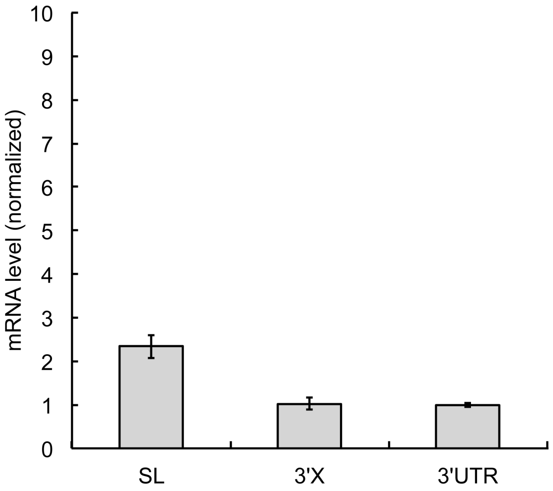

Supplementary Figure 1. The HCV 3'UTR stimulates translation but does not affect mRNA stability. Luciferase RNA levels at the end of the cell based translation assay determined by quantitative RT-PCR. Inclusion of longer 3'end showed no stabilization effect of mRNAs over the course of the experiment. The levels of mRNAs were normalized against the lowest value for comparison purposes. SL indicates the transcript has a 15-nt stem-loop on the 3'end.

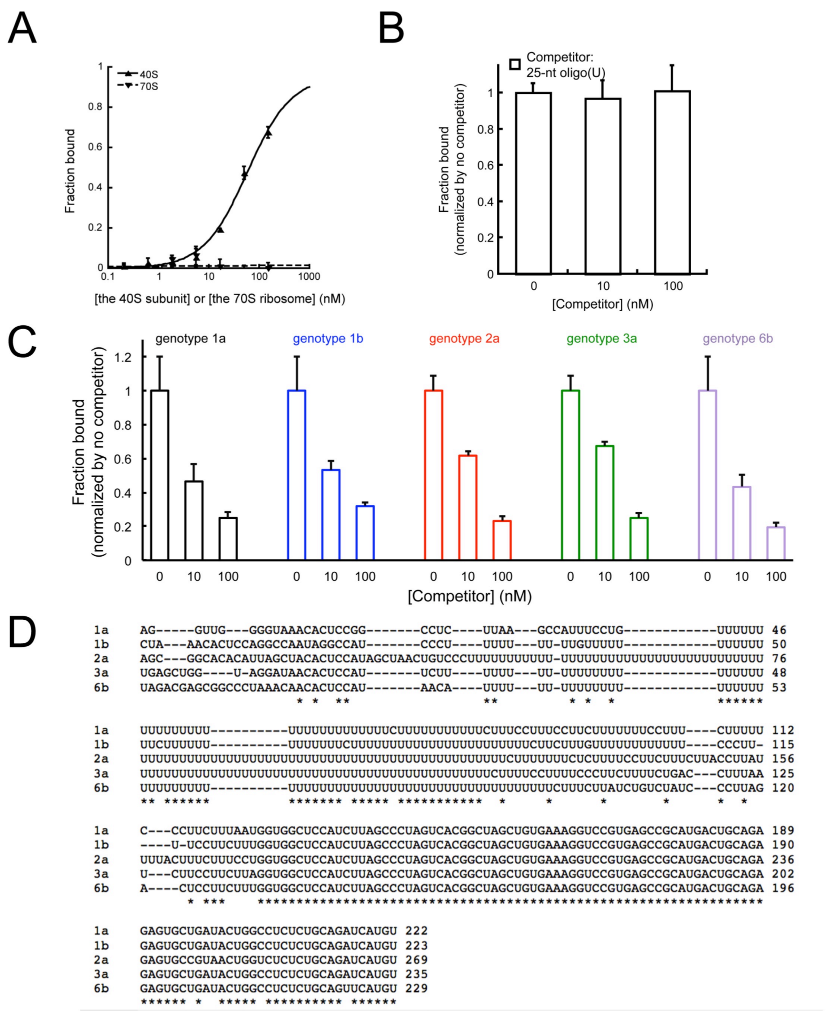

Supplementary Figure 2. The HCV 3'UTR-40S interaction is specific and conserved. **A.** Binding isotherms showing in the presence of 0.1 mg/mL of yeast tRNA, the HCV 3'UTR binds only to the 40S subunit but not the *E. coli* 70S ribosome. **B.** Competition assays showing 25-nt oligo(U) can not compete with the 3'UTR-40S interaction. **C.** Competition assays showing the 3'UTR from different HCV genotypes can compete with each other on binding to the 40S subunit. All competition assays were performed against radiolabeled 3'UTR of HCV genotype 1a. **D.** Sequence alignment for the 3'UTRs from different genotypes. The 3'X region is highly conserved, while the variable region and the length of the poly(U/UC) region varies.

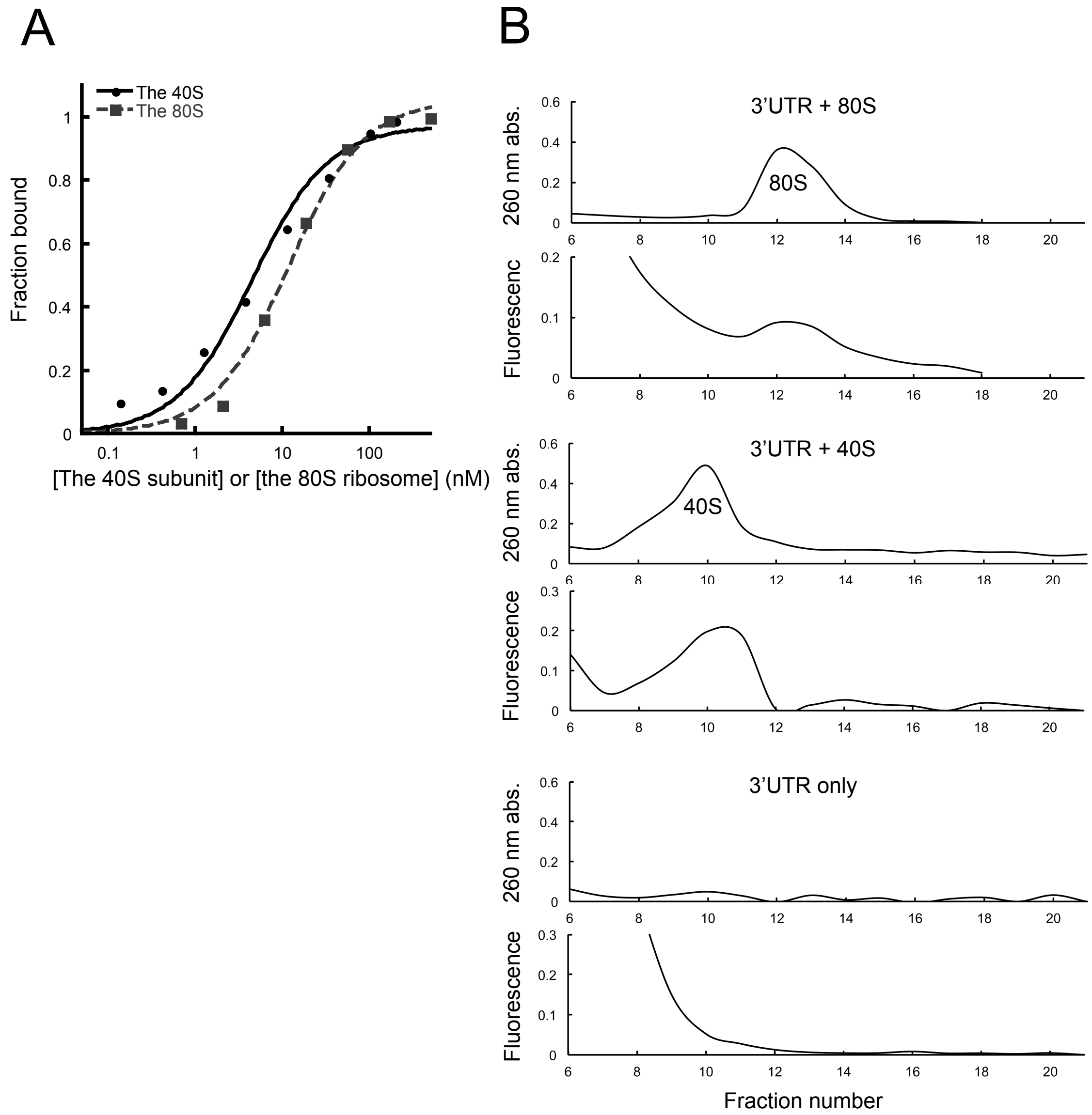

Supplementary Figure 3. The HCV 3'UTR interacts with the 80S ribosome. **(A).** Binding isotherms comparing the interactions of the 3'UTR to 40S subunit and the 80S ribosome. **(B).** Sucrose gradient sedimentation showing the 3'UTR forms complex with both the 40S subunit and the 80S ribosome.

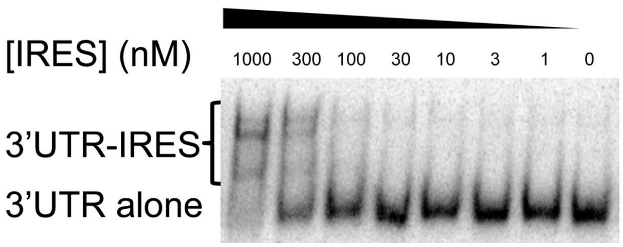

Supplementary Figure 4. The HCV 3’UTR does not have strong interactions with the HCV IRES. Electrophoresis mobility shift assay showing the interactions between the HCV 3’UTR and the HCV IRES. The concentration of radiolabeled HCV 3’UTR is ~ 50 pM. Multiple complex species were observed.

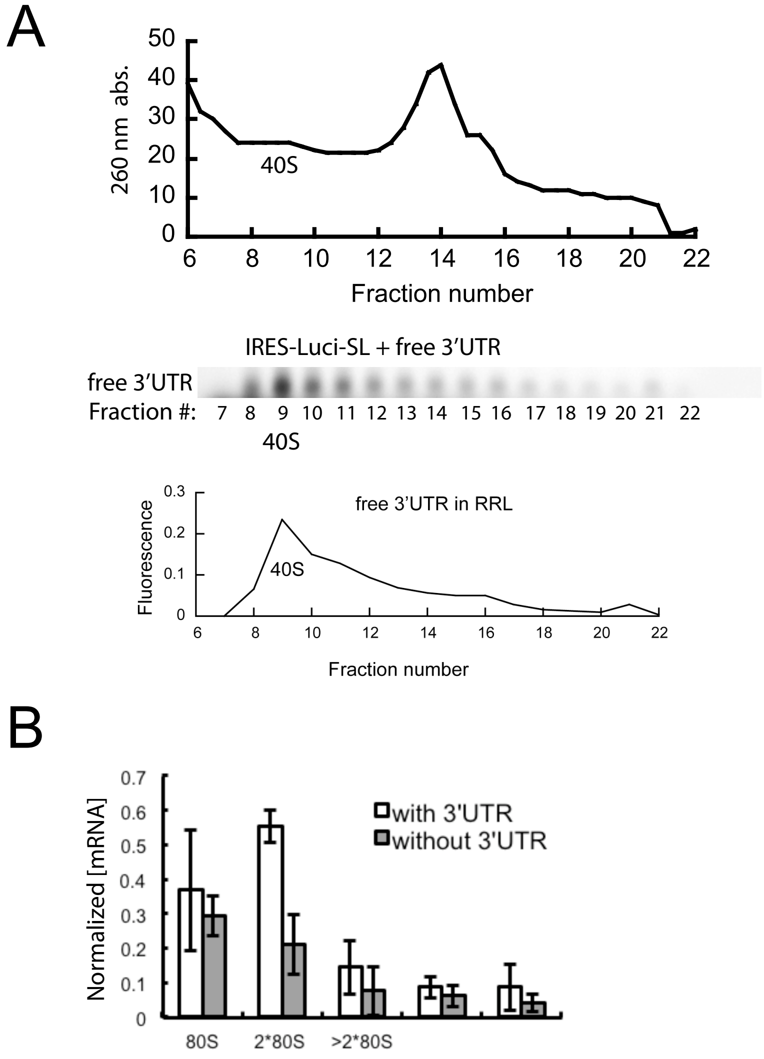

Supplementary Figure 5. HCV 3'UTR promotes polysome formation only *in cis*

A. Sucrose gradient sedimentation results indicated that free 3'UTR added *in trans* to the translation system can co-migrate with the 40S ribosomal subunit. The bottom panel is a quantification of the middle panel. **B.** Sucrose gradient sedimentation results showing that the 3'UTR stimulates formation of heavier polysome in *in vitro* translation reactions.

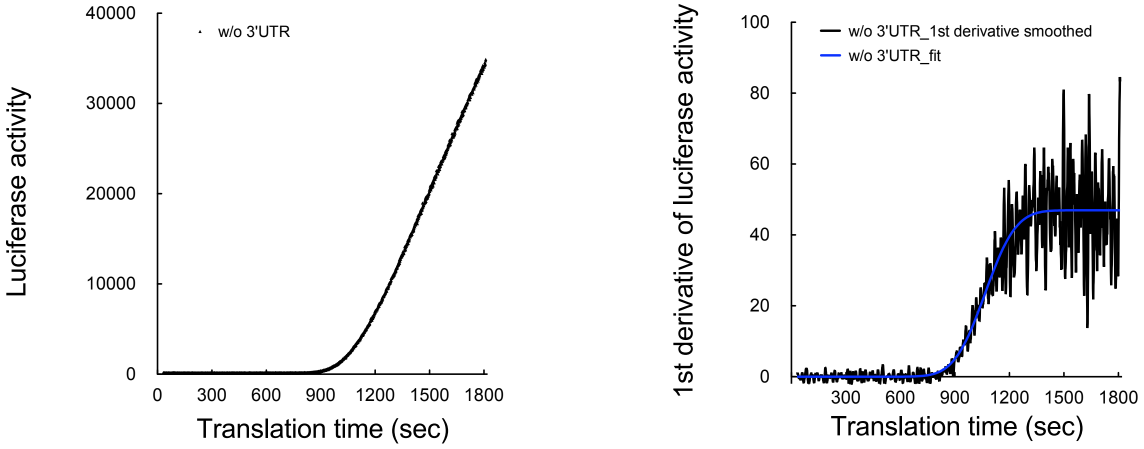

Supplementary Figure 6. Processing of the real-time luciferase activity data. On the left shows the raw data for a real-time luciferase activity measurement. On the right shows the smoothed 1st derivative of the data from the left as well as the curve showing the fitting of the 1st derivative trace to a cumulative distribution function of normal distribution.
